# Qi deficiency constitution increases risk of acute mountain sickness via reduced aerobic fitness

**DOI:** 10.3389/fpubh.2026.1738945

**Published:** 2026-02-16

**Authors:** Shiwei Shen, Yidan Gao, Lingxian Zhu, Shixuan Dai, Mei Tian, Li Jin, Jiucun Wang

**Affiliations:** 1 State Key Laboratory of Genetics and Development of Complex Phenotypes, School of Life Sciences, Fudan University, Shanghai, China; 2 Human Phenome Institute, Zhangjiang Fudan International Innovation Center, Fudan University, Shanghai, China; 3 Ministry of Education Key Laboratory of Contemporary Anthropology, Department of Anthropology and Human Genetics, School of Life Sciences, Fudan University, Shanghai, China

**Keywords:** 3000-meter run performance, acute mountain sickness, aerobic fitness, Qi deficiency constitution, structural equation modeling, traditional Chinese medicine constitution

## Abstract

**Background:**

Acute mountain sickness (AMS) is a common disorder affecting individuals who are exposed to high-altitude environments, generally defined as an elevation of above 2,500 meters. Identifying risk factors for AMS susceptibility before exposure is essential for prevention. According to current research, different constitution types exhibit varying tolerance to acute hypoxia. Exploring the relationship between Traditional Chinese Medicine (TCM) constitution and AMS may therefore provide novel perspectives for prevention and treatment from a TCM standpoint.

**Methods:**

A total of 183 healthy young male participants were enrolled and assessed for TCM constitution, demographic characteristics, clinical indices, and 3,000-meter run performance at low altitude (200 m). After rapid ascent from low altitude to 3,600 m by airplane within 3 h, participants were evaluated for AMS using the Lake Louise Score (LLS). Logistic regression was applied to assess the association between TCM constitution types and AMS, and then linear regression was applied to explore factors associated with Qi deficiency constitution. Structural equation modeling (SEM) was employed to investigate whether aerobic fitness, as reflected by 3,000-meter run performance, mediated the relationship between Qi deficiency constitution and AMS.

**Results:**

The incidence of AMS was 40.4%. Among the nine TCM constitution types, only Qi deficiency was independently associated with an increased risk of AMS (adjusted OR = 1.09, 95% CI: 1.01–1.19, *p* = 0.03). Qi deficiency was significantly associated with 3,000-meter run time, red blood cell count, hemoglobin level, and alcohol intake status. SEM revealed that 3,000-meter run time significantly mediated the association between Qi deficiency and AMS (indirect effect = 0.071, 95% CI: −0.003 to 0.090, *p* = 0.004), while the direct effect was not statistically significant.

**Conclusion:**

Qi deficiency constitution is an independent risk factor for AMS, and this association is mediated through reduced aerobic fitness, reflected by 3,000-meter run performance. Assessing TCM constitution could be a new way to identify individuals at higher risk for AMS before high-altitude exposure. Pre-acclimatization strategies aimed at improving Qi deficiency or enhancing aerobic capacity could help prevent AMS in susceptible populations.

## 1 Introduction

Acute mountain sickness (AMS) is a clinical syndrome that typically presents with symptoms such as headache, dizziness, anorexia, nausea, vomiting, palpitations, and fatigue (1). These symptoms arise following rapid ascent from low-altitude to high-altitude environments (typically above 2,500 m within a short period without adequate acclimatization), primarily due to physiological stress induced by reduced atmospheric pressure, hypobaric hypoxia, lower temperatures, and decreased ambient humidity (2, 3). Although AMS is not fatal in most cases, it can progress to high altitude pulmonary edema (HAPE) and high altitude cerebral edema (HACE), both of which are also known as severe high altitude illness (SHAI) and can be life-threatening (4, 5).

High-altitude regions are generally defined as areas located at elevations exceeding 2,500 meters above sea level, where the risk of developing AMS and other altitude-related illnesses increases significantly (6). China possesses the world’s largest plateau area (7, 8). With the development of plateau tourism and increasing demands for infrastructure projects, the number of individuals entering high-altitude regions has been steadily rising. Consequently, the incidence of high-altitude-related illnesses, particularly AMS, has risen markedly (9–11). This trend underscores the growing importance of research into the pathophysiology, prevention, and management of AMS to safeguard public health and support sustainable development in these regions.

Traditional Chinese Medicine (TCM) defines a constitution as the inherent and relatively stable characteristics of an individual’s morphological structure, physiological function, and psychological state, which develop under the combined influence of genetic endowment and environmental factors (12). TCM constitution is a key factor determining and influencing the occurrence, development, and variation of disease (13). At the same time, it also exhibits susceptibility to certain diseases, as well as a certain tendency toward disease outcomes (14). TCM constitution is divided into Balanced constitution and eight biased constitutions, comprising Qi deficiency constitution, Yang deficiency constitution, Yin deficiency constitution, Phlegm dampness constitution, Damp heat constitution, Qi stagnation constitution, Blood stasis constitution, and Inherited special constitution (15).

The rapid ascent from low-altitude to high-altitude regions triggers various physiological compensatory mechanisms in the human body to adapt to the extreme high-altitude environment (16). Among these, the most critical response is hypoxia-induced stress caused by low atmospheric pressure and reduced oxygen availability (17). Notably, individual physiological responses to hypoxia exhibit significant heterogeneity (18, 19). Previous studies on AMS have primarily focused on environmental factors and conventional physiological indicators, whereas individual susceptibility characteristics remain incompletely understood (20–22). Different types of TCM constitution have varying tolerance to acute hypoxia exposure, as TCM constitution represents a relatively stable, holistic phenotype reflecting an individual’s functional status and stress adaptability, suggesting potential differences in susceptibility to AMS (23). Therefore, this study aimed to investigate the association between AMS and TCM constitution assessed at low altitude. We hypothesized that different TCM constitution types are associated with heterogeneous susceptibility to AMS. Identifying constitution-related differences in AMS susceptibility may provide valuable insights for future prevention and intervention strategies from a TCM perspective.

## 2 Materials and methods

### 2.1 Study population

This study initially recruited 239 healthy young adult males from China, all of whom were first-time entrants to high-altitude environments. The inclusion criteria were as follows: individuals aged 18 years or older, with no history of major diseases such as heart and lung diseases, and no prior exposure to high-altitude areas (approximately 3,600 meters above sea level). Exclusion criteria included a history of neurological or psychiatric disorders, cardiovascular or cerebrovascular diseases, respiratory conditions, chronic headaches, hepatic or renal dysfunction, recent severe upper respiratory infections, or the use of corticosteroids, diuretics, or nonsteroidal anti-inflammatory drugs (NSAIDs) within the past six months.

Due to incomplete data collection, particularly incomplete TCM constitution questionnaire responses, a total of 183 participants were ultimately included in the final analysis. Written informed consent was obtained from all participants prior to the commencement of the study. The research protocol was reviewed and approved by the Ethics Committee of Fudan University (Approval No. FE23023R/233).

### 2.2 Experiment design and sample collection

During the study period, all participants were required to remain at a low-altitude location (approximately 200 meters above sea level, serving as the baseline) for two weeks. During this period, baseline data including age, height, weight, birthplace altitude, ethnicity, level of education, smoking, and alcohol intake status were collected, and clinical biochemical indices such as fasting blood glucose and thyroid function were also collected. Birthplace altitude was determined based on the altitude of the participants’ self-reported birthplace addresses, with elevation obtained by querying the corresponding geographic location. Participants also completed a TCM constitution questionnaire. To assess aerobic fitness, a 3,000-meter run test was administered, and completion times were recorded for subsequent analysis. Following a two-week stay at low altitude, all participants were transported by airplane to a high-altitude region (approximately 3,600 meters above sea level), with the ascent completed within three hours. On the first day after arrival, each participant underwent assessment for AMS using the Lake Louise Score (LLS) questionnaire.

Throughout the study, participants adhered to a standardized diet and abstained from consuming stimulant beverages such as coffee, tea, or alcohol. Daily health checks were conducted by medical personnel to monitor for signs of high-altitude cerebral edema or pulmonary edema. In the event of a medical emergency, immediate clinical intervention was ensured.

#### 2.2.1 Traditional Chinese medicine constitution questionnaire

According to the Chinese national standard issued by the China Association of Chinese Medicine, “Classification and Determination of Traditional Chinese Medicine Constitution standard (ZYYXH/T157-2009)” (24), all participants completed the TCM constitution questionnaire under the supervision of trained personnel certified in TCM.

This standardized instrument categorized individuals into nine constitution types: balanced constitution (considered normal) and eight biased constitutions—Qi deficiency, Yang deficiency, Yin deficiency, Phlegm dampness, Damp heat, Qi stagnation, Blood stasis, and Inherited special constitution. Each constitution type corresponded to a specific subscale comprising 7–8 questions. Each item was scored using a 5-point scale, and the score gradually increased based on the severity of the symptoms. The rating for each question was: 1 point (none), 2 points (rarely), 3 points (sometimes), 4 points (frequently), 5 points (always), with higher scores indicating more prominent constitutional characteristics.

The primary score for each constitution was calculated as the sum of the item scores within its subscale. The calculation formula for the transformed score was:

[(The primary score − number of questions)/(number of questions × 4)] × 100. Each constitution was classified into three categories: “Yes,” “Tendency” and “No.” The classification was based on the transformed score. For the balanced constitution, when its transformed score was ≥60 and all eight biased constitutions were <30, it was judged as “Yes”; while for the eight biased constitutions, when their respective transformed score was ≥40, they were judged as “Yes.”

In this study, because the number of participants meeting the categorical criteria for biased constitutions was relatively small, the primary scores of constitutions were used for subsequent analyses. The full version of the TCM constitution questionnaire is provided in the Supplementary material.

#### 2.2.2 The Lake Louise scoring system

The Lake Louise Scoring System (LLS) is currently the most widely used tool for evaluating AMS (1). The LLS assesses four core symptoms: headache, dizziness, fatigue, and gastrointestinal discomfort. Each symptom is rated on a scale from 0 to 3, where 0 indicates no symptoms; 1, mild symptoms; 2, moderate symptoms that are tolerable; and 3, severe symptoms that are difficult to tolerate. The assessment is conducted on the first day after ascent to high-altitude.

According to the LLS diagnostic criteria (25), a diagnosis of AMS requires the presence of headache (headache score ≥ 1) in combination with a total LLS score of ≥3. Participants who do not meet these criteria are classified as non-AMS.

### 2.3 Statistical analysis

All data processing and statistical analyses were conducted using R software. Missing values for variables such as ethnicity, Education, and smoking status were imputed using median substitution. Categorical variables were expressed as frequencies and percentages [*n* (%)], and comparisons between groups were assessed using the chi-square test. The Shapiro–Wilk test was applied to evaluate the normality of continuous variables. Variables with a normal distribution were presented as mean and standard deviation (SD), and group differences were tested using the independent-sample *t*-test. Non-normally distributed variables were reported as medians and interquartile ranges (IQR), with group comparisons conducted using the Wilcoxon rank-sum test. For correlation analyses, Pearson’s correlation coefficient was used for normally distributed data, and Spearman’s rank correlation coefficient for non-normally distributed data. A two-sided *p*-value < 0.05 was considered statistically significant.

A logistic regression model was employed to investigate the association between AMS and TCM constitution. AMS status (AMS vs. Non-AMS) was used as the dependent variable, and each TCM constitution score as the independent variable. The model was adjusted for potential confounders, including age, BMI, altitude of birthplace, ethnicity, education, smoking and alcohol status. Odds ratios (ORs) and 95% confidence intervals (CIs) were calculated to evaluate the strength and significance of the association. To further identify determinants of the Qi deficiency constitution, multiple linear regression analysis was conducted with the Qi deficiency score as the dependent variable. Independent variables included demographic characteristics, lifestyle factors (smoking and alcohol status), 3,000-meter run time, heart rate, blood pressure, and relevant clinical biochemical indices. Regression coefficients (*β*) and 95% CIs were calculated. Mediation analysis was performed to evaluate whether aerobic fitness, indexed by 3,000-meter run time, mediated the association between Qi deficiency constitution and AMS. Structural equation modeling (SEM) was employed, with AMS treated as a binary outcome. The model was estimated using the weighted least squares mean and variance adjusted (WLSMV) estimator, appropriate for categorical outcomes. Indirect effects were assessed via nonparametric bootstrapping with 5,000 resamples to generate percentile-based 95% CIs. All models were adjusted for age, BMI, altitude of birthplace, ethnicity, education level, smoking, and alcohol status as covariates. Statistical significance was denoted as follows: *p* < 0.05 (*), *p* < 0.01 (**), and *p* < 0.001 (***).

## 3 Results

### 3.1 Incidence of AMS and baseline characteristics of study participants

A total of 183 young adult males were included in the final analysis. Among them, 74 individuals developed AMS, yielding an overall incidence of 40.4%. Baseline characteristics measured at low altitude are summarized in Table 1, while AMS-related outcomes assessed after ascent to high altitude are presented in Table 2.

**Table 1:** Baseline characteristics of study participants.

Characteristics	Study participants	AMS		*p* value
		Non-AMS group	AMS group	
Total number of volunteers, *n* (%)	183	109 (59.6%)	74 (40.4%)	
Age, mean (SD), *y*	19.6 (1.8)	19.8 (1.9)	19.3 (1.5)	0.06
Body Mass Index (BMI), mean (SD), kg/m^2^	23.0 (2.0)	22.9 (2.0)	23.1 (2.1)	0.68
Birthplace altitude, median (IQR), per 100 m	5.9 (2.1–17.7)	12.9 (3.5–19.1)	3.3 (0.9–8.0)	<0.001***
Ethnicity, *n* (%)				0.01*
Han	156 (85.2)	87 (79.8)	69 (93.2)	
Others	27 (14.8)	22 (20.2)	5 (6.8)	
Education, *n* (%)				0.16
College graduate or above	68 (37.2)	36 (33.0)	32 (43.2)	
Less than college graduate	115 (62.8)	73 (67.0)	42 (56.8)	
Smoking status, *n* (%)				0.10
Never/Former	66 (36.1)	34 (31.2)	32 (43.2)	
Current	117 (63.9)	75 (68.9)	42 (56.8)	
Alcohol intake status, *n* (%)				0.27
Never/Former	115 (62.8)	72 (66.1)	43 (58.1)	
Current	68 (37.2)	37 (33.9)	31 (41.9)	
3,000 m running test, median (IQR), min	12.3 (11.6–13.1)	12.3 (11.5–12.5)	12.5 (12.2–13.2)	<0.001***
Heart Rate, median (IQR), bpm	68 (63–73)	67 (62–73)	69 (64–74)	0.18
SBP, mean (SD), mmHg	110.2 (12.9)	109.6 (12.8)	111.0 (13.1)	0.47
DBP, mean (SD), mmHg	63.7 (9.4)	63.6 (8.9)	64.0 (10.1)	0.80
SpO₂, median (IQR), %	99 (97.5–99)	99 (97–99)	99 (98–99)	0.24
Red blood cells, mean (SD),10^12^/L	5.0 (0.3)	5.0 (0.3)	5.1 (0.3)	0.18
Hemoglobin, median (IQR), g/L	153.0 (147.5–158.0)	153.0 (147.0–158.0)	152.0 (149.0–158.0)	0.97
White blood cells, median (IQR), 10^9^/L	6.5 (5.6–7.7)	6.5 (5.5–7.3)	6.8 (5.8–7.8)	0.10
Platelet Count, median (IQR), 109/L	227.5 (202.5–253.5)	224.0 (198.0–251.0)	235.0 (205.2–260.5)	0.07
Fasting blood glucose, mean (SD), mmol/L	4.5 (0.4)	4.5 (0.4)	4.5 (0.4)	0.48
FT3, median (IQR), umol/L	6.0 (5.7–6.4)	6.0 (5.7–6.3)	6.1 (5.8–6.5)	0.16
FT4, median (IQR), umol/L	15.4 (14.3–17.2)	15.3 (14.0–17.1)	15.7 (14.7–17.2)	0.12
TSH, median (IQR), umol/L	2.0 (1.5–2.6)	2.3 (1.6–2.7)	2.0 (1.5–2.4)	0.31

Age, BMI, BP, and fasting blood glucose are presented as mean (SD), and differences between groups were assessed using the independent-sample *t*-test. Birthplace altitude, defined as the altitude of the participants’ self-reported birthplace and expressed per 100 meters, LLS, 3000-meter run time, SpO_2_, red blood cell count, white blood cell count, and thyroid function indicators are presented as median (IQR), with between-group differences tested using the Wilcoxon rank-sum test. Categorical variables, including ethnicity, education level, smoking status, and alcohol intake status, are expressed as *n* (%), and compared using the chi-square test. Statistical significance: *p* < 0.05 (*), *p* < 0.01 (**), *p* < 0.001 (***).

**Table 2:** AMS incidence and outcomes assessed at high altitude.

Characteristics	Non-AMS group	AMS group	*p* value
AMS status, *n* (%)	109 (59.6%)	74 (40.4%)	
Lake Louise score (LLS), median (IQR)	1 (0–2)	3.5 (3–4)	<0.001***

Lake Louise Score was assessed after ascent to high altitude.

As shown in Table 1, the mean age of the study population was 19.6 years, with participants in the AMS group being slightly younger than those in the non-AMS group (19.3 vs. 19.8 years); however, this difference did not reach statistical significance (*p* = 0.06). The median altitude of participants’ birthplace was significantly lower in the AMS group compared with the non-AMS group [330 m vs. 1,290 m; *p* < 0.001], indicating that individuals born at lower altitudes were more likely to develop AMS. In addition, the proportion of Han ethnicity was significantly higher in the AMS group than in the non-AMS group (93.2% vs. 79.8%, *p* = 0.01).

With respect to aerobic fitness, participants in the AMS group demonstrated significantly poorer performance in the 3,000-m running test than those in the non-AMS group [median time: 12.5 min vs. 12.3 min; *p* < 0.001]. No significant differences were observed between the two groups in terms of BMI, education level, smoking status, alcohol intake status, heart rate, blood pressure, peripheral oxygen saturation (SpO₂), hematological parameters, fasting blood glucose, or thyroid function indices.

AMS-related outcomes assessed after ascent to high altitude are summarized in Table 2. As expected, the Lake Louise Score was significantly higher in the AMS group than in the non-AMS group [median (IQR): 3.5 (3–4) vs. 1 (0–2); *p* < 0.001].

### 3.2 Association between TCM constitution and AMS

#### 3.2.1 Relationship between the number of TCM constitution types and AMS

The participants in this study were relatively young (mean age: 19.6 years) and generally healthy, resulting in a small number of individuals with biased constitutions. As shown in Table 3, 11 participants (6% of the cohort) were classified as having a Qi deficiency constitution. In addition, five participants had a Yang deficiency constitution, 10 had a Yin deficiency constitution, four had a Damp heat constitution, 10 had a Phlegm dampness constitution, two had a Qi stagnation constitution, and three had an inherited special constitution. No participants were identified as having a Blood stasis constitution.

**Table 3:** Relationship between the number of TCM constitution types and AMS.

Characteristics	Study participants	AMS	
		Non-AMS group	AMS group
Qi deficiency constitution, *n* (%)			
Qi deficiency	11 (6.0)	7 (6.4)	4 (5.4)
Others (tendency + no)	172 (94.0)	102 (93.6)	70 (94.6)
Yang deficiency constitution, *n* (%)			
Yang deficiency	5 (2.7)	3 (2.8)	2 (2.7)
Others (tendency + no)	178 (97.3)	106 (97.2)	72 (97.3)
Yin deficiency constitution, *n* (%)			
Yin deficiency	10 (5.5)	8 (7.3)	2 (2.7)
Others (tendency + no)	173 (94.5)	101 (92.7)	72 (97.3)
Phlegm dampness constitution, *n* (%)			
Phlegm dampness	4 (2.2)	2 (1.8)	2 (2.7)
Others (tendency + no)	179 (97.8)	107 (98.2)	72 (97.3)
Damp heat constitution, *n* (%)			
Damp heat	10 (5.5)	5 (4.6)	5 (6.8)
Others (tendency + no)	173 (94.5)	104 (95.4)	69 (93.2)
Blood stasis constitution, *n* (%)			
Blood stasis	0 (0)	0 (0)	0 (0)
Others (tendency + no)	183 (100)	109 (100)	74 (100)
Qi stagnation constitution, *n* (%)			
Qi stagnation	2 (1.1)	0 (0)	2 (2.7)
Others (tendency + no)	181 (98.9)	109 (100)	72 (97.3)
Inherited special constitution, *n* (%)			
Inherited special	3 (1.6)	1 (0.9)	2 (2.7)
Others (tendency + no)	180 (98.4)	108 (99.1)	72 (97.3)
Balanced constitution, *n* (%)			
Balanced	60 (32.8)	30 (27.5)	30 (40.5)
Others (tendency + no)	123 (67.2)	79 (72.5)	44 (59.5)

Each constitution was classified into three categories: “Yes,” “Tendency” and “No”; Others included both “Tendency” and “No.” The number of participants for each constitution type is expressed as counts and percentages.

Given the limited number of participants classified into the eight biased TCM constitution types, categorical comparisons across constitution subgroups were statistically unstable. Such small cell sizes may lead to biased *p*-values and imprecise effect size estimates. Consequently, the primary constitution scores were analyzed as continuous variables rather than categorical classifications in subsequent analyses, thereby improving statistical power and yielding a more robust evaluation of the association between TCM constitution and AMS.

#### 3.2.2 Correlation between TCM constitution scores and LLS

In this study, a correlation analysis was conducted between the primary scores of TCM constitution and the LLS recorded on the first day after arrival at high altitude. As shown in Table 4, the balanced constitution was negatively correlated with LLS, while the other eight biased constitutions exhibited positive correlations. Among them, the Qi deficiency constitution showed the strongest correlation with LLS (*r* = 0.31, *p* < 0.001), indicating a statistically significant association. Similarly, the Inherited special constitution (*r* = 0.20, *p* = 0.005), Yang deficiency constitution (*r* = 0.17, *p* = 0.01), and Phlegm dampness constitution (*r* = 0.16, *p* = 0.02) also demonstrated significant correlations. Although Yin deficiency, Damp heat, Qi stagnation, and Blood stasis constitutions were positively correlated with LLS, these correlations were weaker and did not reach statistical significance.

**Table 4:** Correlation between TCM constitution scores and LLS.

TCM constitution scores	Correlation coefficient (*r*)	*p* value
Qi deficiency constitution	0.31	<0.001***
Yang deficiency constitution	0.17	0.01*
Yin deficiency constitution	0.13	0.07
Phlegm dampness constitution	0.16	0.02*
Damp heat constitution	0.11	0.12
Blood stasis constitution	0.05	0.45
Qi stagnation constitution	0.10	0.16
Inherited special constitution	0.20	0.005**
Balanced constitution	−0.06	0.40

TCM constitution scores were non-normally distributed. Spearman’s rank correlation coefficients were calculated to assess the association between constitution scores and LLS. Statistical significance: *p* < 0.05 (*), *p* < 0.01 (**), *p* < 0.001 (***).

#### 3.2.3 Relationship between TCM constitution scores and AMS

As presented in Table 5, in the AMS group, the median (IQR) score of the Qi deficiency constitution was 14 (11–17), while in the non-AMS group was 12 (10–15). The Qi deficiency score in the AMS group was higher than that in the non-AMS group, and the difference between two groups was statistically significant (*p* = 0.003). No statistically significant differences were observed between the AMS and non-AMS groups in the other constitution types, including Yang deficiency, Yin deficiency, Phlegm dampness, Damp heat, Blood stasis, Qi stagnation, Inherited special constitution, or Balanced constitution.

**Table 5:** Differences in TCM constitution scores between AMS and non-AMS.

TCM constitution scores	Total participants	AMS		*p* value
		Non-AMS group	AMS group	
Qi deficiency constitution, median (IQR)	13 (10–16)	12 (10–15)	14 (11–17)	0.003**
Yang deficiency constitution, median (IQR)	9 (8–11.5)	9 (7–11)	9 (8–13)	0.26
Yin deficiency constitution, median (IQR)	12 (10–15)	12 (10–16)	13 (10–15)	0.59
Phlegm dampness constitution, median (IQR)	11 (9–13)	11 (9–12)	11 (9–13)	0.20
Damp heat constitution, median (IQR)	9 (7–11)	9 (7–11)	9 (7–11)	0.17
Blood stasis constitution, median (IQR)	9 (8–11)	9 (8–11)	9.5 (8–11)	0.49
Qi stagnation constitution, median (IQR)	8 (7–10)	8 (7–10)	8 (7–10)	0.41
Inherited special constitution, median (IQR)	9 (7–11)	9 (7–11)	9 (8–11)	0.06
Balanced constitution, median (IQR)	35 (32–38)	36 (32–38)	35 (32–38)	0.65

TCM constitution scores are presented as median (IQR). Differences between the AMS and non-AMS group were assessed using the Wilcoxon rank-sum test. Statistical significance: *p* < 0.05 (*), *p* < 0.01 (**), *p* < 0.001 (***).

#### 3.2.4 Logistic regression analysis of TCM constitution scores and AMS

To further assess the relationship between TCM constitution and AMS, logistic regression was performed with AMS status (non-AMS vs. AMS Group) as the dependent variable and the scores of the nine TCM constitution as independent variables.

As shown in Table 6, Model 1 is a univariate logistic regression, indicating that among the nine TCM constitution, only Qi Deficiency constitution was significantly associated with AMS (OR = 1.08, 95% CI: 1.01–1.17, *p* = 0.02), suggesting that higher Qi Deficiency scores were linked to an increased risk of AMS. No significant associations were observed for the other constitution types.

**Table 6:** Logistic regression results of TCM constitution and AMS.

TCM constitution	Model 1		Model 2	
	OR (95%CI)	*p* value	OR (95%CI)	*p* value
Qi deficiency constitution	1.08 (1.01, 1.17)	0.02*	1.09 (1.01, 1.19)	0.03*
Yang deficiency constitution	1.04 (0.95, 1.13)	0.44	1.02 (0.91,1.11)	0.85
Yin deficiency constitution	1.00 (0.93, 1.08)	0.93	1.02 (0.94, 1.11)	0.64
Phlegm dampness constitution	1.05 (0.96, 1.15)	0.29	1.06 (0.96, 1.18)	0.24
Damp heat constitution	1.06 (0.96,1.16)	0.24	1.09 (0.98, 1.21)	0.12
Blood stasis constitution	1.04 (0.93, 1.17)	0.48	1.15 (1.00, 1.32)	0.05
Qi stagnation constitution	0.98 (0.88, 1.09)	0.77	1.00 (0.89, 1.13)	0.95
Inherited special constitution	1.09 (0.98, 1.22)	0.10	1.13 (0.99, 1.28)	0.06
Balanced constitution	0.99 (0.91, 1.07)	0.79	0.97 (0.88, 1.06)	0.47

Model 1: univariate logistic regression. Model 2: multivariate logistic regression adjusted for age, BMI, altitude of birthplace, ethnicity, education level, smoking and alcohol status. Statistical significance: *p* < 0.05 (*), *p* < 0.01 (**), *p* < 0.001 (***).

**Figure 1:**
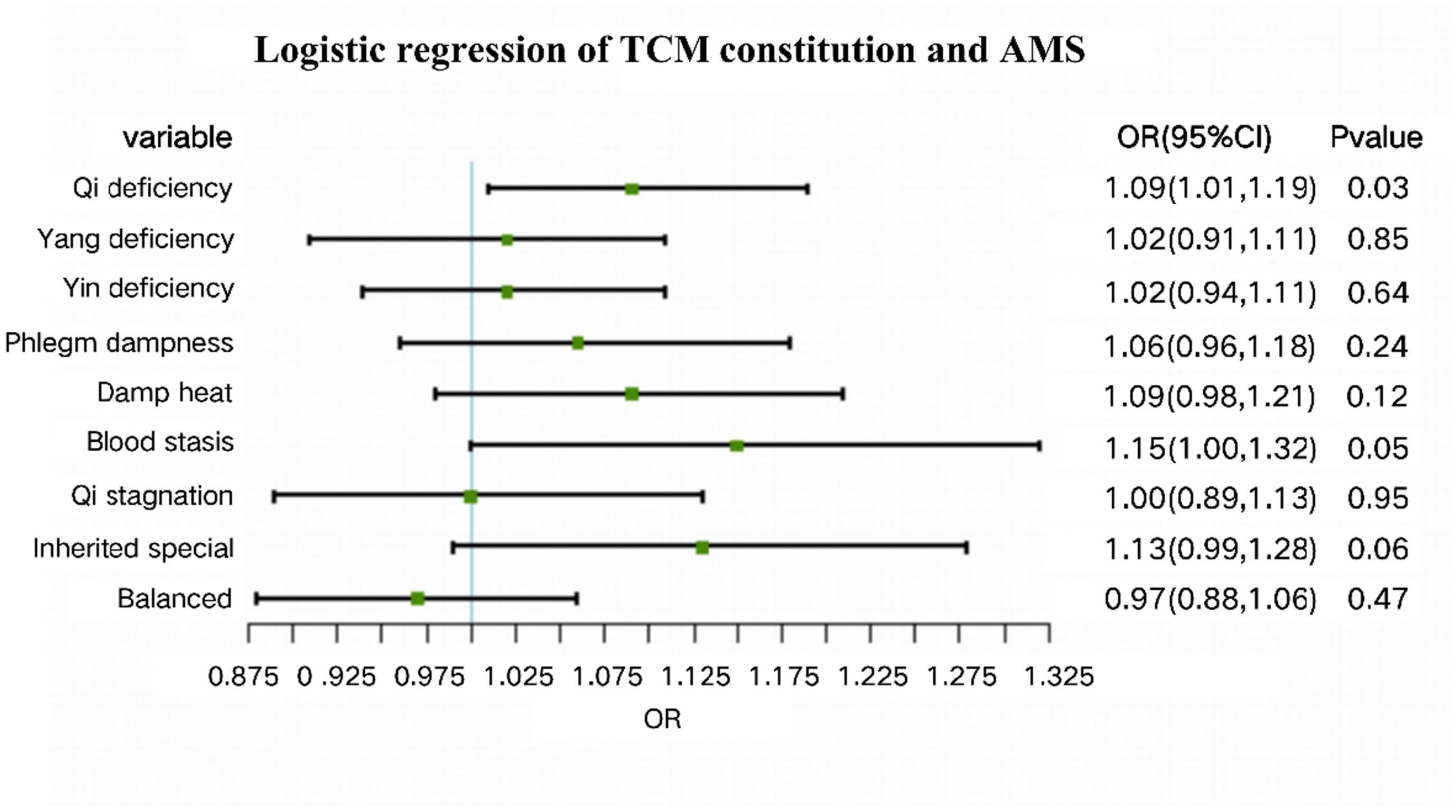
Forest plot of logistic regression results for TCM constitution and AMS.

In Model 2, as shown in Figure 1, multivariate logistic regression was conducted with adjustments for potential confounders, including age, BMI, altitude of birthplace, ethnicity, education level, and smoking and alcohol status. As Qi Deficiency constitution was the only significant factor in Model 1, the other constitution types were not included in the adjusted model. The results confirmed Qi Deficiency constitution as an independent risk factor for AMS (OR = 1.09, 95% CI: 1.01–1.19, *p* = 0.03). Blood stasis constitution (OR = 1.15, 95% CI: 1.00–1.32, *p* = 0.05) and Inherited special constitution (OR = 1.13, 95% CI: 0.99–1.28, *p* = 0.06) showed borderline associations with AMS, but these did not reach conventional levels of statistical significance. No significant associations were found for Yang deficiency, Yin deficiency, Phlegm dampness, Damp heat, Qi stagnation, or Balanced constitution.

This forest plot presents the OR (95% CIs) for the association between the scores of nine TCM constitution types and AMS. An OR greater than 1 indicates increased risk, whereas an OR less than 1 suggests a potential protective association. Qi deficiency constitution was significantly associated with a higher risk of AMS after adjustment for confounders.

### 3.3 Multivariate linear regression analysis of factors associated with Qi deficiency constitution

As shown in Table 7, in Model 1, which was based on univariate linear regression, current alcohol drinkers were significantly associated with higher Qi deficiency scores compared to non-current drinkers (*β* = 1.65; 95% CI: 0.42–2.88; *p* = 0.009). Poorer aerobic fitness, reflected by longer 3,000-meter run time, was also strongly correlated with higher Qi deficiency scores (*β* = 1.23; 95% CI: 0.60–1.86; *p* < 0.001). No other individual variables, including birthplace altitude, ethnicity, smoking status, heart rate, blood pressure, white blood cell count, red blood cell count, hemoglobin level, fasting blood glucose, or thyroid function, showed statistically significant associations in the unadjusted models.

**Table 7:** Multiple linear regression analysis of factors associated with Qi deficiency constitution score.

Variable	Model 1	*p* value	Model 2	*p* value
	*β* (95% CI)		*β* (95% CI)	
Demographics				
Age (years)	−0.01 (−0.35 to 0.34)	0.96	−0.02 (−0.40 to 0.37)	0.94
BMI (kg/m^2^)	0.26 (−0.04 to 0.56)	0.09	0.12 (−0.19 to 0.44)	0.44
Ethnicity (Han)	1.13 (−0.57 to 2.83)	0.19	1.00 (−1.11 to 3.10)	0.35
Birthplace altitude (per 100 m)	0.003 (−0.06 to 0.06)	0.92	0.02 (−0.06 to 0.10)	0.64
Education level (college graduate or above)	−0.04 (−1.29 to 1.22)	0.96	−0.12 (−1.44 to 1.19)	0.85
Lifestyle				
Smoking status (current)	−0.13 (−1.39 to 1.13)	0.85	−0.51 (−1.77 to 0.75)	0.42
Alcohol intake status (current)	1.65 (0.42 to 2.88)	0.009**	1.39 (0.12 to 2.65)	0.03*
Fitness and vital signs				
3,000 m running test (minutes)	1.23 (0.6 to 1.86)	<0.001***	1.20 (0.49 to 1.91)	0.001**
Heart rate (bpm)	0.04 (−0.03 to 0.11)	0.24	0.03 (−0.05 to 0.10)	0.47
SBP (mmHg)	−0.02 (−0.07 to 0.02)	0.35	−0.07 (−0.07 to 0.05)	0.81
DBP (mmHg)	−0.03 (−0.09 to 0.04)	0.39	−0.02 (−0.10 to 0.06)	0.69
Hematology				
Red blood cells (×10^12^/L)	1.29 (−0.68 to 3.27)	0.20	2.64 (0.11 to 5.17)	0.04*
Hemoglobin (g/L)	−0.04 (−0.11 to 0.02)	0.17	−0.09 (−0.17 to −0.005)	0.04*
White blood cells (×10^9^/L)	0.31 (−0.08 to 0.7)	0.11	0.32 (−0.09 to 0.72)	0.13
Platelet count (×10^9^/L)	0.002 (−0.01 to 0.02)	0.79	−0.01 (−0.03 to 0.006)	0.24
Fasting blood glucose (mmol/L)	−0.04 (−1.72 to 1.64)	0.96	−0.18 (−1.88 to 1.51)	0.83
FT3 (umol/L)	−0.16 (−1.19 to 0.87)	0.76	−0.37 (−1.53 to 0.78)	0.52
FT4 (umol/L)	0.18 (−0.08 to 0.44)	0.17	0.08 (−0.21 to 0.38)	0.58
TSH (umol/L)	−0.16 (−0.66 to 0.34)	0.54	−0.34 (−0.84 to 0.17)	0.19

Model 1: univariate linear regression. Model 2: multivariable linear regression including all listed covariates. Ethnicity was dichotomized as Han vs. Others; education level as college graduate or above vs. less than college graduate; smoking status and alcohol intake as current vs. non-current (never/former). No evidence of multicollinearity was found in Model 2 (all VIF < 5). Statistical significance: *p < 0.05; **p < 0.01; ***p < 0.001.

In Model 2, a multivariable linear regression was conducted including demographic variables, smoking and alcohol status, 3,000-meter run time, heart rate, blood pressure, and laboratory indicators. The results showed that alcohol drinkers were significantly associated with higher Qi deficiency scores compared with non-current drinkers (*β* = 1.39; 95% CI: 0.12–2.65; *p* = 0.03). The 3,000-meter run time also remained significantly associated with Qi deficiency; participants with longer performance had higher scores (*β* = 1.20; 95% CI: 0.49–1.91; *p* = 0.001). Additionally, red blood cell and hemoglobin level became statistically significant only after adjusting for covariates. Higher Qi deficiency score was associated with higher red blood cell count (*β* = 2.64; 95% CI: 0.11–5.17; *p* = 0.04) and lower hemoglobin levels (*β* = −0.09; 95% CI: −0.17 to −0.005; *p* = 0.04). Other variables, including blood pressure, heart rate, fasting glucose, and thyroid function, were not significantly associated with Qi deficiency constitution in the multivariable model.

### 3.4 Mediation effect of 3,000-meter run performance in the association between Qi deficiency constitution and AMS

Although red blood cell count, hemoglobin, and current alcohol drinkers were significantly associated with Qi deficiency scores, none of these factors showed a significant association with AMS in previous analyses. In contrast, 3,000-meter run performance, as an objective indicator of aerobic capacity, was significantly associated with both Qi deficiency and AMS. Specifically, individuals with higher scores of Qi deficiency were more likely to exhibit poorer performance in a 3,000-meter run, which in turn increased the risk of developing AMS. Since Qi deficiency constitution reflects a subjective, symptom-based assessment, while the 3,000-meter run time provides an objective, performance-based measure of physical function, the latter was regarded as a plausible mediator. To formally test this hypothesis, a mediation analysis was conducted using a SEM framework to evaluate whether aerobic fitness mediates the relationship between Qi deficiency constitution and AMS.

**Figure 2:**
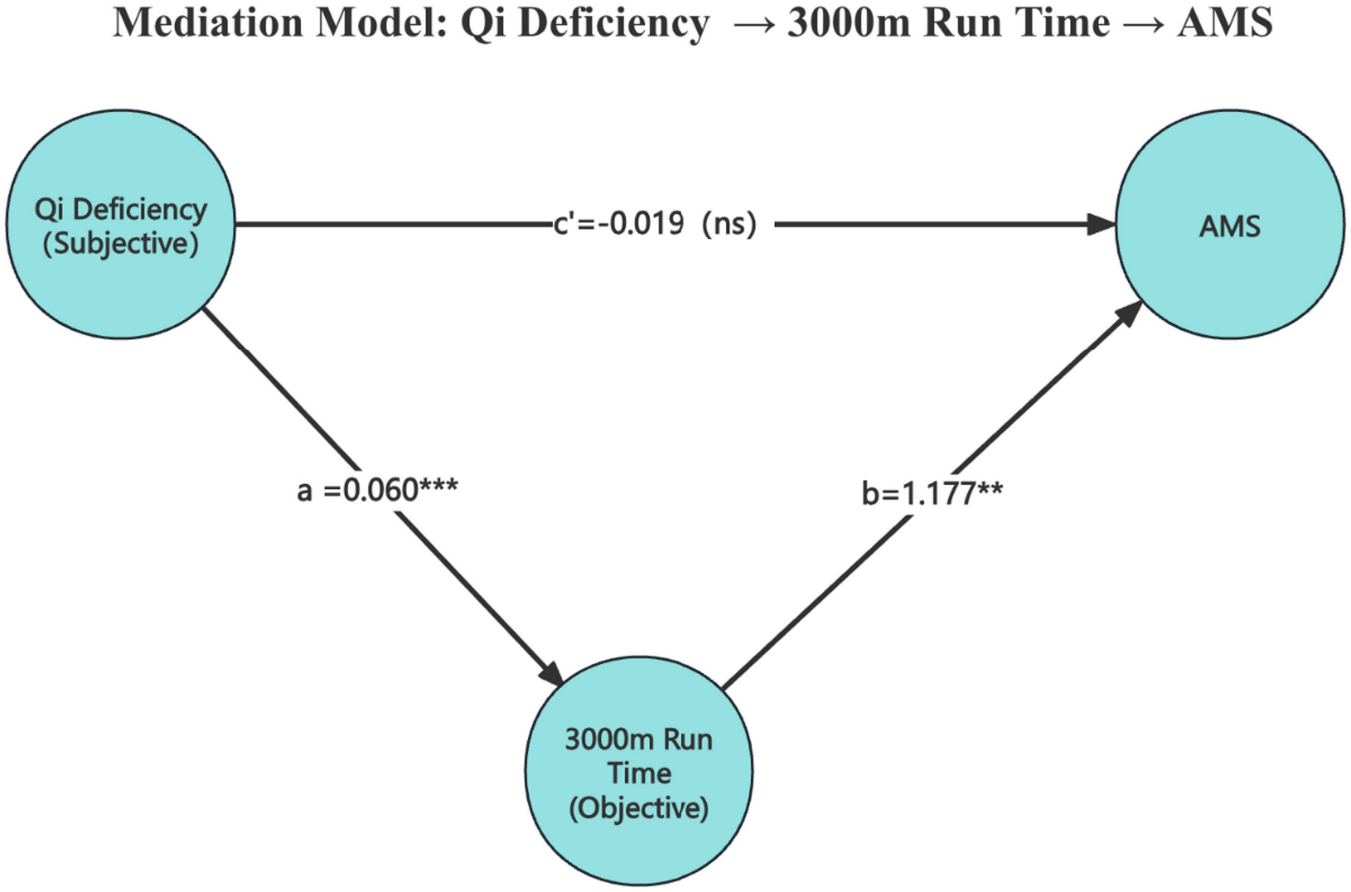
Mediation model of the association among Qi deficiency constitution, 3,000-meter run performance, and AMS.

As shown in Table 8 and Figure 2, mediation analysis was conducted to examine whether 3,000-meter run performance mediated the relationship between Qi deficiency constitution and AMS. The analysis revealed that higher Qi deficiency scores were significantly associated with slower 3,000-meter run times (*a path*: *β* = 0.060, SE = 0.016, 95% CI = 0.027–0.091, *p* < 0.001). In turn, poorer 3,000-meter run performance was positively associated with an increased risk of AMS (*b path*: *β* = 1.177, SE = 0.398, 95% CI = −0.050 to 1.297, *p* = 0.003). The indirect effect of Qi deficiency on AMS through 3,000-meter run performance was statistically significant (*β* = 0.071, SE = 0.025, 95% CI = −0.003 to 0.090, *p* = 0.004). In contrast, the direct effect of Qi deficiency on AMS was not significant (*c′ path*: *β* = −0.019, SE = 0.039, 95% CI = −0.054 to 0.108, *p* = 0.624). The total effect combining both direct and indirect pathways showed a marginal association (*β* = 0.052, SE = 0.029, 95% CI = 0.004–0.119, *p* = 0.076).

**Table 8:** Mediation analysis of the relationship among Qi deficiency constitution, 3,000-meter run performance and AMS.

Path/effect	Estimate	SE	95% CI	*p* value
*a path*: Qi deficiency →3,000 m run time	0.060	0.016	0.027 to 0.091	<0.001***
*b path*: 3,000 m run time → AMS	1.177	0.398	−0.050 to 1.297	0.003**
*c′ path*: Qi deficiency → AMS (direct)	−0.019	0.039	−0.054 to 0.108	0.624
Indirect effect (*a* × *b*)	0.071	0.025	−0.003 to 0.090	0.004**
Total effect (*c′* + *a* × *b*)	0.052	0.029	0.004 to 0.119	0.076

Mediation analysis of the relationship among Qi deficiency constitution, 3,000-meter run performance, and AMS. Results are presented as estimates with standard errors (SE) and 95% confidence intervals (CI) based on 5,000 bootstrap resamples. *a path* refers to Qi deficiency → 3,000-meter run time; *b path* refers to 3,000-meter run time → AMS; *c*′ *path* refers to the direct effect of Qi deficiency on AMS. The indirect effect is calculated as *a* × *b*, and the total effect as *c*′ + *a* × *b*. Statistical significance: *p < 0.05; **p < 0.01; ***p < 0.001.

These findings suggest that the relationship between Qi deficiency constitution and AMS may be explained, at least in part, by impaired aerobic fitness as reflected by 3,000-meter run performance, supporting its role as a functional mediator in this pathway.

## 4 Discussion

In recent years, there has been a growing trend of individuals traveling to high-altitude regions for tourism, work, athletic training, or military deployment (26). Therefore, early recognition of risk factors and identification of susceptible individuals at low altitude is critical to reducing the incidence of AMS (27).

Of the 183 participants in this study, 74 developed AMS, yielding an incidence rate of 40.4%, which is consistent with previous studies and may be attributable to the rapid ascent to high altitude by air travel (21, 28). We observed that participants born at lower altitudes had a higher risk of AMS compared to those born at higher elevations. Additionally, Han ethnicity was associated with a greater incidence of AMS relative to ethnic minorities, such as Tibetans. These findings suggest that individuals born at high altitudes may have developed enhanced physiological adaptations to hypoxia, likely due to genetic and long-term environmental exposure, which reduce their susceptibility to AMS (29, 30). These findings underscore the importance of birthplace altitude and ethnic background as contributors to AMS susceptibility.

Meanwhile, our findings revealed the 3,000-meter run test conducted at low altitude, as an indicator of aerobic capacity, was significantly associated with the risk of AMS. The 3,000-meter run is a widely used field test for evaluating cardiorespiratory fitness and is considered a practical method for estimating maximal oxygen uptake (VO_2_ max) (31, 32). VO_2_ max reflects an individual’s maximum capacity to transport and utilize oxygen. A higher VO_2_ max is associated with superior exercise capacity and better cardiovascular health (33, 34). Participants who developed AMS exhibited poorer running performance, possibly due to reduced cardiorespiratory fitness and insufficient physiological reserves, which may increase susceptibility to hypoxic environments at high altitude and thereby elevate the risk of AMS (35). This observation is consistent with several previous studies demonstrating that lower aerobic fitness is associated with an increased risk of AMS (35, 36). In contrast, other physiological indicators such as heart rate, blood pressure, hematologic parameters, and thyroid hormone levels did not show significant associations with AMS in this cohort.

Qi deficiency constitution emerged as an independent risk factor for AMS. The Qi deficiency constitution score showed the strongest correlation with the LLS, and participants in the AMS group had significantly higher Qi deficiency score compared to those in the non-AMS group. Logistic regression analysis further confirmed that the Qi deficiency constitution was an independent risk of AMS even after adjusting for confounding factors. Qi deficiency constitution is one of the most common deficiency constitutions in TCM, ranking second among unbalanced constitutions in the Chinese population and accounting for approximately 13.18% of individuals (37). Previous studies have suggested that Qi deficiency is closely associated with impaired cardiovascular and pulmonary function, including clinical conditions such as chronic obstructive pulmonary disease (COPD) and chronic heart failure (38, 39). Meanwhile, Qi deficiency is characterized by symptoms such as fatigue, shortness of breath, and decreased exercise tolerance, which are consistent with reduced aerobic capacity (40, 41). Considering that individuals with Qi deficiency constitution may have relatively weak compensatory ability in cardiovascular and pulmonary function, and have poor tolerance to hypoxia, they are prone to AMS after exposure to high-altitude environments.

Qi deficiency may be the underlying pathophysiological basis of susceptibility to AMS. Many Chinese herbal medicines used for the intervention of AMS have Qi-tonifying effects, such as Huangqi Baihe granules, salidroside, and ginsenoside Rg1 (7). Ginsenoside Rg1, for example, has been shown to improve oxygen utilization, reduce inflammation, and protect vascular function under hypoxic conditions (42). Other studies have identified modulation of the HIF-1α/NF-κB signaling pathway and reduction in oxidative stress as potential mechanisms underlying these effects (7). These findings indirectly support the notion that Qi deficiency is an important underlying pathophysiological factor in AMS, as individuals with Qi deficiency constitution are more prone to exhibiting Qi deficiency syndromes and thus more susceptible to AMS under hypoxic exposure.

Aerobic fitness was further identified as a potential mediator linking Qi deficiency constitution to AMS. Although Qi deficiency constitution was identified as an independent risk factor for AMS in the present study, the physiological mechanisms underlying this association remain incompletely understood. Our study revealed that Qi deficiency constitution was associated with several physiological and hematological indicators. In multivariable linear regression, longer 3,000-meter run time and current alcohol drinkers were both significantly associated with higher Qi deficiency scores. Participants with poorer 3,000-meter run performance, a proxy measure for aerobic capacity, tended to have higher Qi deficiency scores, suggesting that these individuals may have diminished cardiopulmonary endurance. In addition, red blood cell (RBC) count and hemoglobin levels showed significant associations in adjusted models, with Qi deficiency scores positively correlated with RBC count and negatively correlated with hemoglobin concentration. These findings suggest that Qi deficient individuals may have an underlying state of mild tissue hypoxia, which triggers erythropoietic responses without sufficient hemoglobin synthesis or utilization, indicating a possible compensatory hematologic response to subclinical hypoxia (43–45).

However, although RBC count, hemoglobin level, and current alcohol drinking status were significantly associated with Qi deficiency scores, none of these factors showed significant association with AMS. Notably, 3,000-meter run performance was significantly associated with both Qi deficiency and AMS. Therefore, a structural equation modeling approach was applied to examine the relationship among Qi deficiency constitution, 3,000-meter run performance, and AMS. The results revealed that 3,000-meter run time partially mediated the association between Qi deficiency and AMS. Specifically, the indirect effect was statistically significant, whereas the direct effect was not. These results indicate that aerobic fitness, as reflected by 3,000-meter run performance, plays a mediating role in the pathway linking Qi deficiency to AMS susceptibility (46–48).

The 3,000-meter run is a validated field measure of maximal oxygen consumption (VO₂ max), representing the integrated capacity of the cardiovascular, respiratory, and muscular systems to deliver and utilize oxygen (31, 32). VO₂ max, defined as the maximum capacity of the cardiovascular, respiratory, and muscular systems to deliver and utilize oxygen, is a key indicator of cardiopulmonary function and is clinically relevant in conditions such as heart failure (HF), hypertrophic cardiomyopathy (HCM), and chronic obstructive pulmonary disease (COPD) (49, 50). It is widely regarded as the gold standard for assessing cardiopulmonary fitness (51, 52). Existing studies have shown that individuals who developed AMS at 3900 m had significantly lower VO₂ max compared to those without AMS (35). These findings highlight the importance of cardiopulmonary fitness in high-altitude acclimatization. Individuals with prominent Qi deficiency may exhibit reduced cardiopulmonary capacity and diminished physiological resilience to hypoxic stress, which could impair their ability to acclimatize to high-altitude environments. Collectively, our results suggest that reduced physical endurance may represent a key physiological pathway linking Qi deficiency constitution to heightened vulnerability to AMS during acute hypoxic exposure.

## 5 Limitation

This study has several limitations: First, the study population consisted exclusively of young Chinese males, which may limit the generalizability of the findings to females, older individuals, or other ethnic groups. However, the use of a relatively homogeneous cohort may have reduced potential confounding related to sex- and age-dependent physiological variability. Second, TCM constitution assessment was based on a standardized but self-reported questionnaire, which may be subject to inherent subjectivity and measurement variability. Accordingly, constitution scores were analyzed as continuous variables to better reflect the spectrum of constitutional characteristics within a relatively homogeneous population, rather than relying solely on categorical classification. Third, although our SEM analysis identified a statistically significant indirect pathway through aerobic capacity, this approach does not exclude the possibility that other unmeasured physiological or behavioral mechanisms may also contribute, which should be investigated in future studies.

## 6 Conclusion

This study suggests that Qi deficiency constitution is an independent risk factor for AMS, and that this association is mediated through reduced aerobic fitness. These findings integrate TCM constitution with modern physiological markers, offering a holistic and practical framework for AMS risk assessment and prevention. From a clinical prevention perspective, pre-acclimatization strategies that improve Qi deficiency constitution through the use of Qi-tonifying herbal medicine or by enhancing aerobic fitness may help reduce the risk of AMS in susceptible populations.

## Data Availability

The original contributions presented in the study are included in the article/Supplementary material, further inquiries can be directed to the corresponding author/s.

## Glossary

TCM: traditional Chinese medicine
AMS: acute mountain sickness
LLS: lake Louise Score
SEM: structural equation modeling
HAPE: high altitude pulmonary edema
HACE: high altitude cerebral edema
SHAI: severe high altitude illness
NSAIDs: nonsteroidal anti-inflammatory drugs
SD: standard deviation
IQR: interquartile ranges
ORs: odds ratios
CIs: confidence intervals
WLSMV: weighted least squares mean and variance adjusted
COPD: chronic obstructive pulmonary disease
VO_2 max_: field measure of Maximum oxygen consumption
HF: heart failure
HCM: hypertrophic cardiomyopathy
SBP: systolic blood pressure
DBP: diastolic blood pressure
FT3: free triiodothyronine
FT4: free thyroxine
TSH: thyroid stimulating hormone
