# Supplementary material for: Qi deficiency constitution increases risk of acute mountain sickness via reduced aerobic fitness
Source: Front Public Health. 2026 Feb 16;14:1738945. doi: 10.3389/fpubh.2026.1738945 (PMC12950716; doi:10.3389/fpubh.2026.1738945)
Supplement: Supplementary file 1 [file Table_1.docx]

**Supplementary Materials**

**Table S1. The standard of classification and determination of Traditional Chinese Medicine Constitution**

| **Constitution** |  | **Constitution questions** |
| --- | --- | --- |
| Yang  Deficiency  Constitution  (YDC) | Q1 | Did your hands and feet feel cold? |
|  | Q2 | Did you feel cold easily in your abdomen, back, lower back or knees? |
|  | Q3 | Were you sensitive to the cold and tend to wear more clothes than others? |
|  | Q4 | Did you feel more vulnerable to the cold (cold in winter, cold air conditioning in summer, electric fans, etc.) than others? |
|  | Q5 | Did you catch colds more easily than others? |
|  | Q6 | Did you feel uncomfortable or afraid of eating (drinking) cold things? |
|  | Q7 | Are you prone to diarrhea (diarrhea) after catching cold or eating (drinking) cold things? |
| Yin Deficiency  Constitution  (YINDC) | Q1 | Did the palms of your hands or soles of your feet feel hot? |
|  | Q2 | Did your body and face feel hot? |
|  | Q3 | Did your skin or lips feel dry? |
|  | Q4 | Were your lips redder than others? |
|  | Q5 | Did you get constipated easily or have dry stools? |
|  | Q6 | Did you get hot flashes? |
|  | Q7 | Did your eyes feel dry and use eye drops? |
|  | Q8 | Did you often feel parched and need to drink water? |
| Qi Deficiency  Constitution  (QDC) | Q1 | Did you get tired easily? |
|  | Q2 | Did you suffer from shortness of breath? |
|  | Q3 | Did you get palpitations easily? |
|  | Q4 | Did you get dizziness easily or become giddy when standing up? |
|  | Q5 | Did you catch colds more easily than others? |
|  | Q6 | Did you prefer quietness and do not like to talk? |
|  | Q7 | Did you feel weak when talking? |
|  | Q8 | Did you sweat easily when you had a slightly increased physical activity? |
| Phlegm  Dampness  Constitution  (PDC) | Q1 | Did you feel chest or stomach stuffiness? |
|  | Q2 | Did your body feel heavy or lethargic? |
|  | Q3 | Was your belly plump and flabby? |
|  | Q4 | Did you have an excessively oily forehead? |
|  | Q5 | Did you have upper eyelid swelling? |
|  | Q6 | Did your mouth feel sticky? |
|  | Q7 | Did you have lots of phlegm, especially in your throat? |
|  | Q8 | Did your tongue have a thick coating? |
| Damp Heat  Constitution  (DHC) | Q1 | Did your nose or your face feel greasy, oily, or shiny? |
|  | Q2 | Did you get acne or sores easily? |
|  | Q3 | Did you have bitterness or a strange taste in your mouth? |
|  | Q4 | Did you pass sticky stools and/or feel that your bowel movement is incomplete? |
|  | Q5 | Did your urethral canal feel hot when you urinated，or did your urine have a dark color? |
|  | Q6 | Was your vaginal discharge yellowish(only for female interviewees)? |
|  | Q7 | Was your scrotum always wet (only for male interviewees)? |
| Blood Stasis  Constitution  (BSC) | Q1 | Did black or purple bruises appear on your skin for no reason? |
|  | Q2 | Did you have visible capillary/thread veins on your cheeks? |
|  | Q3 | Did you feel pain somewhere in your body? |
|  | Q4 | Did you have a dull complexion or get melasma easily? |
|  | Q5 | Did you get dark circles under the eyes easily? |
|  | Q6 | Did you forget things easily? |
|  | Q7 | Did your lips darker, more blue or purple than others? |
| Qi Stagnation  Constitution  (QSC) | Q1 | Did you feel gloomy and depressed? |
|  | Q2 | Did you get anxious and worried easily? |
|  | Q3 | Did you feel sensitive，vulnerable or emotionally upset? |
|  | Q4 | Were you easily scared or frightened? |
|  | Q5 | Did you experience distention in the underarm or breast? |
|  | Q6 | Did you sigh for no reason? |
|  | Q7 | Did your throat feel strange(i.e., like something was stuck or there was a lump in your throat)? |
| Inherited  Special  Constitution  (ISC) | Q1 | Did you sneeze even when you did not have a cold? |
|  | Q2 | Did you have runny or stuffy nose even when you did not have a cold? |
|  | Q3 | Did you cough due to seasonal change，temperature change，or unpleasant odor? |
|  | Q4 | Did you have allergies?(E.g. medicine, food, odors, pollen, pet dander, or during seasonal or weather change etc.)? |
|  | Q5 | Did your skin get hives/urticaria easily? |
|  | Q6 | Did your skin have purpura (purple spots, ecchymosis) due to allergies? |
|  | Q7 | Did your skin turn red and show traces when you scratched it? |
| Balanced  Constitution  (BC) | Q1 | Were you energetic? |
|  | Q2 | Could you adapt yourself to external natural or social environment change? |
|  | Q3 | Did you suffer from insomnia? |
|  | Q4 | Did you get tired easily? |
|  | Q5 | Did you feel weak when talking?* |
|  | Q6 | Did you feel gloomy and depressed?* |
|  | Q7 | Did you feel more vulnerable to the cold (cold in winter, cold air conditioning in summer, electric fans, etc.) than others?* |
|  | Q8 | Did you forget things easily?* |

| Scoring | |
| --- | --- |
| Single question scoring criteria | Each question is scored on a 5-point scale, with 1 point being awarded for None, 2 points for Rarely, 3 points for Sometimes, 4 points for Often, and 5 points for Always. |
| Original score  calculation | The scores of each item are added up (note: questions marked * are scored in reverse  order, for example: 1 -> 5, 2 -> 4, 3 -> 3, 4 -> 2, 5 -> 1) |
| Transformation score  calculation | [(the original score - number of items) / (number of items * 4)] * 100 |

| **Classification standards** | | |
| --- | --- | --- |
| **Constitution**  **type** | **Classifi**  **cation** | **Scoring** |
| Balanced  constitution | Yes | The transformation score is ≥ 60 points and the transformation scores of the other eight  pathological constitutions are all < 30 points. |
|  | Maybe | The transformation score is ≥ 60 points and the transformation scores of the other eight  pathological constitutions are all < 40 points. |
|  | No | Not meet the above conditions |
| Biased constitution | Yes | The transformation score is ≥ 60 points |
|  | Maybe | The transformation score is ≥ 30 points and < 40 points |
|  | No | The transformation score is < 30 points |
